# Developments of Cyanobacteria for Nano-Marine Drugs: Relevance of Nanoformulations in Cancer Therapies

**DOI:** 10.3390/md16060179

**Published:** 2018-05-23

**Authors:** Vivek K. Bajpai, Shruti Shukla, Sung-Min Kang, Seung Kyu Hwang, Xinjie Song, Yun Suk Huh, Young-Kyu Han

**Affiliations:** 1 Department of Energy and Materials Engineering, Dongguk University-Seoul, 30 Pildong-ro 1-gil, Seoul 04620, Korea; vbiotech04@gmail.com (V.K.B.); shrutishukla1983@gmail.com (S.S.); 2 WCSL of Integrated Human Airway-on-a-chip, Department of Biological Engineering, Biohybrid Systems Research Center (BSRC), Inha University, 100 Inha-ro, Nam-gu, Incheon 22212, Korea; sungmin.kang21@gmail.com (S.-M.K.); h3418kr@gmail.com (S.K.H.); 3 Department of Food Science and Technology, Yeungnam University, Gyeongsan-si, Gyeongsangbuk-do 38541, Korea

**Keywords:** microalgae/cyanobacteria, nanoformulation, drug development, commercial drawbacks

## Abstract

Current trends in the application of nanomaterials are emerging in the nano-biotechnological sector for development of medicines. Cyanobacteria (blue-green algae) are photosynthetic prokaryotes that have applications to human health and numerous biological activities as dietary supplements. Cyanobacteria produce biologically active and chemically diverse compounds such as cyclic peptides, lipopeptides, fatty acid amides, alkaloids, and saccharides. More than 50% of marine cyanobacteria are potentially exploitable for the extraction of bioactive substances, which are effective in killing cancer cells by inducing apoptotic death. The current review emphasizes that not even 10% of microalgal bioactive components have reached commercialized platforms due to difficulties related to solubility. Considering these factors, they should be considered as a potential source of natural products for drug discovery and drug delivery approaches. Nanoformulations employing a wide variety of nanoparticles and their polymerized forms could be an emerging approach to the development of new cancer drugs. This review highlights recent research on microalgae-based medicines or compounds as well as their biomedical applications. This review further discusses the facts, limitations, and commercial market trends related to the use of microalgae for industrial and medicinal purposes.

## 1. Introduction

The potential of marine life as a source of novel drug molecules is immense and has recently garnered interest for commercialization purposes. In particular, microalgae are a potential source of useful drug products containing polyunsaturated fatty acids, vitamins, lipids, proteins, and a variety of bioactive compounds. Some of the specific advantages of microalgae cultivation compared to traditional plant-based sources include a faster cultivation, processing, and harvesting cycle as well as the ability to be cultured on waste materials, which enhance drug cost effectiveness and biopotential. Furthermore, isolated compounds, extracts, and fractioned extracts of microalgae have been reported to possess important biological activities, including anti-cancer, anti-inflammatory, antioxidant, microbicidal, anti-leprosy, and leishmanicidal activities, as well as to reduce triacylglyceride levels in the liver and serum [1]. Therefore, many studies have been published on microalgae, and many patents for chemicals extracted from marine algae have been registered. 

Despite many recent advances in cancer treatment, cancer malignancy remains the fundamental cause of death worldwide. According to the National Cancer Institute, tumors kill 171.2 for every 100,000 individuals annually [2]. Cancer is one of the most widespread and feared diseases largely due to its difficulty to cure, eventually resulting in death. The main reason for this difficulty is that cancer results from the uncontrolled multiplication of subtly modified normal human cells. Around 975,396 new cancer cases and 358,392 cancer-associated deaths occurred among young adults worldwide in 2012, which equates to an age-standardized rate of 43.3 new cancer cases per 100,000 people per year and 15.9 cancer-associated deaths per 100,000 people per year [3].

In the current global market, a variety of anti-cancer drugs are available across a wide cost range. Comparison of the cost of the 10 highest earning cancer drugs between the USA and Norway has elucidated severe differences in both the price and affordability of these drug agents [4]. Treatment modalities for cancer include surgery (for solid cancer), radiotherapy, and chemotherapy [5]. This third category of treatment is mediated by therapeutic molecules with anti-tumor activity. Anti-cancer drugs are mostly based on alkylating agents and anti-metabolites, which inhibit DNA synthesis [5].

Numerous microalgae-derived compounds have been systematically tested for their biomedicinal and therapeutic potentials against cancer [6,7]. Microalgae-based natural components provide a broad platform for cancer treatments, and there has been scientific research on algae-based anti-cancer components [8]. Despite difficulties in collecting marine samples, a huge number of marine samples have gained approval for use in the pharmaceutical market as health beneficial supplements [9]. 

However, due to a shortcoming of corresponding ethnomedical evidences along with technical problems associated with marine flora collection strategies, progressive developments in marine algae-based compounds as therapeutic molecules compared with their terrestrial counterparts are still ongoing. Over the past several years, a huge amount of effort has been paid by various pharmaceutical and research collaborations to isolate and characterize new bioactive molecules from marine flora, particularly from microalgal species [10]. In spite of such effort, marine flora has remained unexplored to a certain extent, and these works are reviewed here as baseline data for promoting further research in this field.

Many natural compound-based drugs against cancer have been discovered, but they have poor water solubility, which makes their formulation difficult or even impossible [11]. For example, camptothecin is widely recognized as an efficient anti-cancer agent in vitro, but its clinical application is limited due to its poor solubility [12,13]. 

To address these difficulties, nanotechnology can provide a novel method to overcome the poor water solubility of hydrophobic natural marine drugs. The field of nanomedicine has achieved significant advances in the use of nanocarrier formulations for delivering therapeutic drugs and diagnostic agents to tumor/cancer sites. Compared with the systemic administration of free natural bioactive molecules, utilization of nanomedicines presents unique advantages in terms of improved protection of the biological activities of the agents in a serum-rich environment, prolonged circulation periods in the bloodstream, reduced adverse effects, enhanced permeability and retention effects, improved tumor-targeting efficiency, increased release profiles, and possible integration of stimuli-responsiveness for on-demand therapeutics, among others [11,13,14,15]. Encapsulation of hydrophobic drugs into nanoparticles (<200 nm) can render natural algae-based drugs completely dispersible in water, making the drugs intravenously injectable. After drug-loaded nanoparticles are administrated, the drug can be released from nanoparticles and can play a role as a disease inhibitor [13]. 

**Figure 1:**
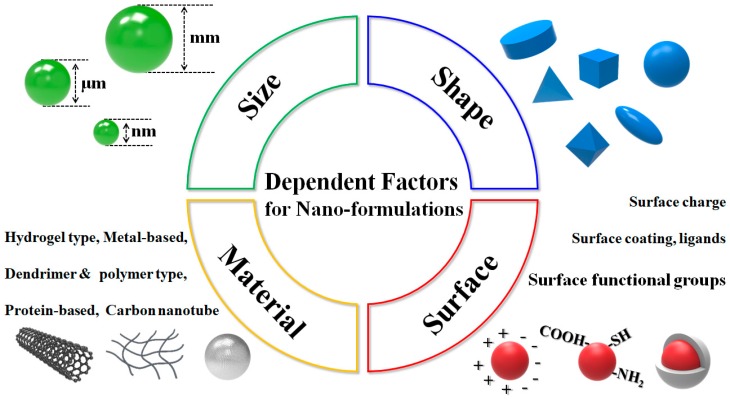
Overview of regulative parameters of nanoparticles use (biomaterials, metal and metal-oxides, carbon-based) for devising an optimized nanoformulation for clinical drug applications.

Nanoformulations of natural drugs derived from marine algae can be designed using various nanoparticles with favorable properties for facilitating delivery in a variety of cancer situations [13]. For example, nanoparticles can be fabricated from materials of various origin, including both inorganic (e.g., metals, silica, carbon, and their respective oxides) and organic (e.g., polymers and lipids). Further, their sizes can be manipulated to fall within a wide range, from a few nanometers to no more than 1 mm, their shapes can be tuned to be smooth or sharp, their plasticity can be altered to be stiff or transformable, and their surfaces can be functionalized with many different characteristics and moieties of interest (Figure 1). 

Another advantage of nanoformulations composed of natural cancer components is their versatility and ability to rationally design nanoparticles to overcome sequential biological barriers (e.g., sequestration by the mononuclear phagocytic system, MDR), which have been shown to limit the efficacy of nanoparticle-based drug delivery [16,17]. Indeed, the first nanoparticles developed were shown to passively accumulate in tumors through an enhanced permeability and retention (EPR) effect [18,19,20]. 

**Figure 2:**
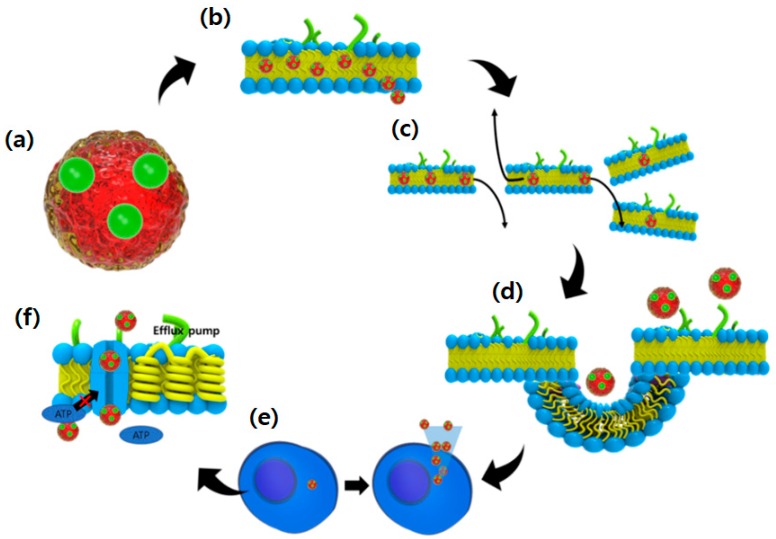
The advantages of using nanoparticles for delivering microalgal anticancer agents. (**a**) improved solubility of anticancer agents; (**b**) enhanced circulation time of anticancer agents in the blood vessels; (**c**) facilitation of the accumulation of anticancer agents in targeted tumor tissues; (**d**) targeting features of nanoparticles allow drug uptake by tumor cells through endocytosis, resulting in increased intracellular drug concentrations; (**e**) achieve controlled and stable drug release; and (**f**) minimization of efflux pump-mediated drug-resistance since nanoparticles are not substrates for ATP-binding cassette proteins.

The paramount advantages of nanoparticles carrying anticancer agents of interest is that they can enhance drug efficacy by a number of fascinating ways, such as (a) improved solubility for better drug delivery; (b) increased half-life in circulation, due to resistance to the reticuloendothelial system or mononuclear phagocytic system (MPS); (c) enhanced drug accumulation in target cancer tissues or cells; (d) constant and stable drug release; and (e) reduced efflux pump-mediated drug-resistance (Figure 2).

These findings have increased our interest in the availability of microalgae-based natural bioactive anti-cancer compounds and commercialized microalgal drugs. Here, microalgae-based drugs in nanoformulations, their future perspectives, and possible therapeutic strategies are discussed. Also, a new strategy for the development of microalgae-based bioactive anti-cancer nanoformulations with polymerization is presented for the development and commercialization of cost effective, stable, and efficient anti-cancer drugs as an alternative to expensive cancer therapies. By developing microalgal bioactive nanoformulation drugs, it will be possible to build data sets for marine-based pharmaceuticals. Translatable clinical development and patient’s pre-selection strategies will help these effective therapies reach the right patients via intravenous and/or oral administration.

## 2. Microalgal Uniqueness: Recent Paths to Anti-Cancer Compound Discovery

Marine diversity includes a vast microbial flora (bacteria, actinobacteria, cyanobacteria, and fungi), microalgae, macroalgae (seaweeds), and flowering plants (mangroves or halophytes). Microalgae are a diverse group of single-cell photosynthetic organisms [21]. They can be either a prokaryote (cyanobacteria) or a eukaryote, by growing in different ecological environments and producing diverse metabolites [22,23]. The three most important classes of microalgae in terms of abundance are the diatoms (*Bacillariophyceae*), the green algae (*Chlorophyceae*), and the golden algae (*Chrysophyceae*). The cyanobacteria or blue-green algae (*Cyanophyceae*) are also referred to as microalgae, i.e., *Spirulina* (*Arthrospira platensis* and *Arthrospira maxima*) [24,25]. With a view of rich oceanic biodiversity, the micro-flora along with microalgae constitutes more than 90% of the oceanic biomass [26]. This unique availability of microalgae offers great potential for the discovery of new drugs. 

Importantly, 50% of marine blue-green algal species are cultivated in commercial platforms to extract bioactive compounds, which have shown enormous potential to kill a variety of cancer cells via induction of apoptosis, or by affecting the activation of cell signaling enzymes, especially the protein kinase-c family members [27,28]. Moreover, depending on the species and environment flora, marine microalgae contain huge amounts of various types of proteins, dietary fiber, polyunsaturated fatty acids (PUFAs), vitamins and minerals [27,28].

Among these compounds, polysaccharides extracted from microalgae play a principal role as anti-cancer agents in commercialized platforms. Scytonemin, a well-known commercialized extracellular pigment derived from a cyanobacterium *Scytonema* has shown great ability to regulate the formation of mitotic spindles and enzyme kinase, having a direct association in the control of the cell cycle as well as showing inhibitory potential against the cell proliferation of human endothelial and fibroblast cells [29]. In addition, scytonemin, from cyanobacterium *Lyngbya* sp. U2555 was characterized and proven for its induction by UV radiation, and stability under different abiotic factors [30].

The calothrixins are quinone-based natural products isolated from *Calothrix* cyanobacteria that have shown potent antiproliferative effects against several cancer cell lines [31]. Interestingly, calothrixin B has been found to display antiproliferative activity against HCT-116 colon cancer cell line with IC_50_ value of 0.32 µM [32]. Some microalgae-based compounds have shown enormous potential to inhibit a variety of colon cancer cells. For example, malyngamides are small amides produced by marine cyanobacteria; malyngamides such as malyngamide C and 8-epi-malyngamide C isolated from *Lyngbya majuscule* were found to be cytotoxic to HT29 colon cancer cells with IC_50_ value of 5.2 and 15.4 μM, respectively [33]. Merocyclophanes A and B, isolated from a terrestrial *Nostoc* sp. (UIC 10022A), displayed antiproliferative activity against HT-29 cell line with IC_50_ value of 3.3 and 1.7 µM, respectively [34]. Further, hierridin B, a secondary metabolite (polyketide) isolated from the marine cyanobacterium *Cyanobium* sp. LEGE 06113, tested in a panel of eight human cancer cell lines, showed selective cytotoxicity against colon cancer cell line HT-29 with an IC_50_ value of 0.1 mM [35]. Furthermore, a cyanobacterial peptide glembatumumab vedotin approved for Phase II trials has shown potent efficacy for the treatment of breast and melanoma cancers with the maximum tolerant doses of 1.0–1.88 mg/kg [36]. 

Apratoxins, a class of cyclic depsipeptides originally isolated from a marine cyanobacterium from Micronesia, exhibited high potency against various cancer cells [37]. Apratoxin A was shown to induce pronounced G cell cycle arrest and apoptosis. Moreover, apratoxin A was shown to reversibly inhibit the secretory pathway by preventing co-translational translocation [38]. 

Konickova et al. [39] previously reported a few bilirubin-like compounds from microalgal sources such as *Spirulina platensis* and *S. platensis*-derived tetrapyrroles using an experimental model of pancreatic cancer. In addition to this, largazole isolated from *Symploca* sp. with a unique chemical structure has shown significant antiproliferative activity [40]. Apratoxins are another class of cyanobacterial compounds that inhibit a variety of cancer cell lines at nano-molar concentration. Apratoxin A (parental compound), isolated from a cyanobacterial strain *Lyngbya boulloni* has been found to exhibit cytotoxicity against adenocarcinoma cells [41]. A compound coibamide A isolated from *Leptolyngbya* sp. [42], was reported to be toxic against NCIH460 lung and mouse neuro-2a cells. Cytotoxicity is a common mechanism of action of several cyanobacterial compounds [43]. In this context, the most significant discoveries are of borophycin, cryptophycin 1 and 8, and cyanovirin. Borophycin is a boron-containing metabolite, isolated from marine cyanobacterial species of *Nostoc linckia* and *N. spongiaeforme* var. *tenue* [44]. The compound exhibits potent cytotoxicity against human epidermoid carcinoma (LoVo) and human colorectal adenocarcinoma (KB) cell lines [45].

**Figure 3:**
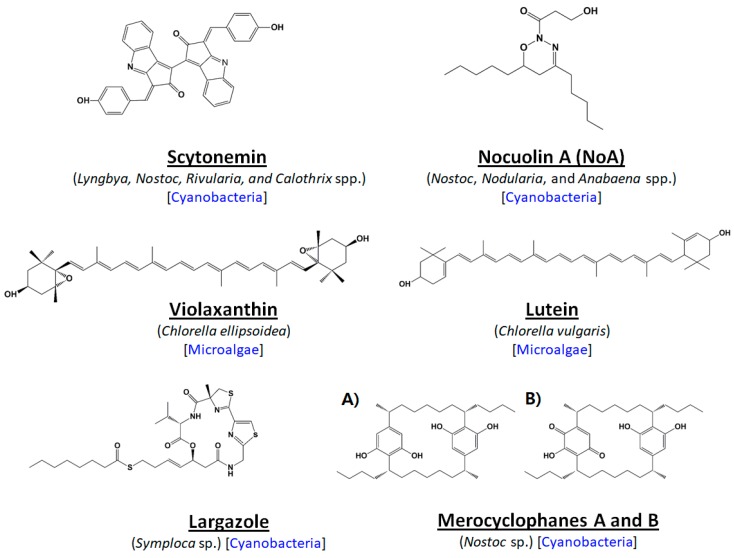
List of selected anticancer compounds derived from cyanobacteria or microalgae for effective nanoformulation.

Moreover, extracts from *Chlorella vulgaris*, whose main constituent was lutein, displayed anti-proliferative effects on a human colon cancer cell line (HCT-116) [46]. More recently, lutein has shown chemo-protective efficacy against colorectal cancer in an animal model by modulating the proliferative activity of K-ras, protein kinase B (PKB) and β-catenin [47]. Furthermore, extracts from the marine microalga *Chlorella ellipsoidea*, possessing violaxanthin as its major component, have shown potent antiproliferative effects on a human colon cancer cell line (HCT-116) by inducing apoptosis [46]. Also, violaxanthin was found to display pro-apoptotic and antiproliferative activities against human cancer cell lines [48]. The chemical structures of a few selected compounds isolated from microalgae for effective nanoformulation are given in Figure 3.

Specific cyanobacterial genera such as *Nostoc*, *Nodularia*, and *Anabaena* have been found to possess unique biodiversity of natural products, as confirmed by the isolation of a natural oxadiazine nocuolin A (NoA) from some of the species belonging to these genera with a unique 1,2,3-oxadiazine structure, and detailing the clusters of the putative genes for the biosynthesis of NoA in their genomes [49]. It was reported that NoA-induced cell death has the same attributes as caspase-dependent apoptosis. Interestingly, NoA was found to exhibit anti-proliferative effects against a number of human cancer cells, more specifically against p53-mutated cell lines within a IC_50_ value ranging from 0.7–4.5 μM [49]. 

NoA is a novel class of natural metabolite with a heterocyclic structure. Similar heterocyclic structures were previously reported only in synthetic compounds, mostly based on 1,3,4- or 1,2,4-oxadiazines, where the N–N–O system is interrupted by a carbon atom incorporated either between two nitrogen atoms or a nitrogen and an oxygen atom [50]. The actual N–N–O linkage was reported solely in five-membered rings, i.e., 1,2,3-oxadiazole derivatives [51]. The existence of a closed 1,2,3-oxadiazole ring was initially reported to be improbable since an open ring form is more stable, as predicted by quantum chemical modelling [52]. However, the equilibrium can be shifted towards the cyclic form by introducing adequate substituents, as demonstrated in many synthetic 1,2,3-oxadiazole derivatives [53]. The discovery of NoA brings new insights into the chemistry of heterocyclic structures containing N–N–O linkage as well as their natural occurrence. From the pharmaceutical perspective, the 1,3,4-oxadiazoles in particular represent a considerable platform for drug development [50]. Since oxadiazole structures were demonstrated to exhibit a broad range of biological activity, there is a strong interest in developing new 1,3,4-oxadiazole derivatives. Synthetic 1,3,4-oxadiazine derivatives manifested high efficiencies against cancer cells in nano-molar range [54]. 

Furthermore, cancer cells possess p53 mutations being resistant to a variety of conventional cancer therapeutic agents, thus the efficacy of NoA could be accounted in cancer treatment by targeting tumor cells independent of their p53 position, thereby offering an ideal strategy in cancer drug development. In brief, the hypothesis for the anticancer mechanistic pathways adopted by various anticancer drugs, including metallic nanoparticles (NPs) is a fundamental understanding of apoptosis induced by these agents, such as silver (Ag) nanoparticles and thus their subsequent use would depend on their mode of function [55]. Basic investigations have shown cytotoxic stress-induced cellular function disrupting ability of NPs leading to the damage of cell membrane. In this context, the role of various signaling genes has been ascertained in programmed cell death through the induction of apoptosis in mammalian cells by the interaction of AgNPs with mammalian cells leading to fragmentation of cellular DNA via p53 dependent mitochondrial signaling pathway [56]. Several studies of the cytotoxicity of AgNPs on mammalian cells have confirmed apoptosis as their primary mode of action, involving a direct role of reactive oxygen species (ROS)-mediated oxidative stress in DNA damage and/or mitochondrial-dependent apoptosis pathway [57]. In addition, regulation (up and/or down) of pro- and anti-apoptotic members of Bcl-2 gene family has an important role in the activation of apoptosis caspases, which are known to play a vital role in both initiation and execution of apoptosis [56]. To this end, the role of caspase3 was confirmed in cellular DNA fragmentation previously [58]. Since AgNP was found to upregulate the expression of caspase-3 gene in BHK21 and HT29 cells, it could be suggested that these compounds could be mediators of apoptosis leading to programmed cell death [56].

**Figure 4:**
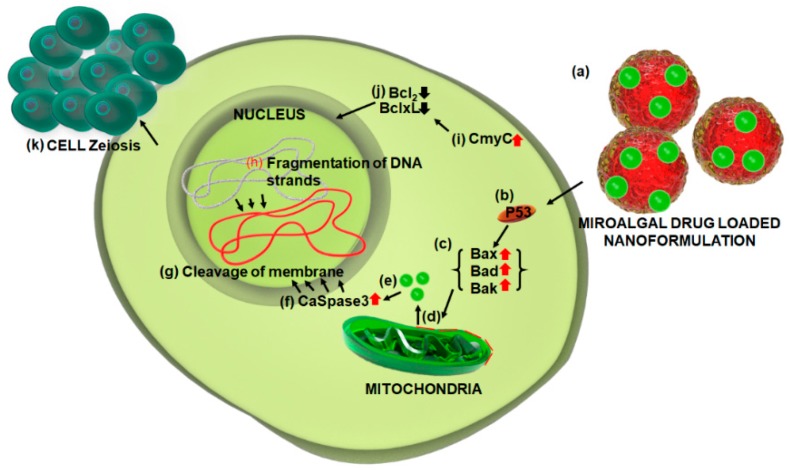
Possible molecular mechanism of microalgal drug loaded nanoformulation in cancer therapy via mitochondrial-induced apoptotic pathway. (**a**) Attachment od microalgal drug loaded nanoformulation to cell membrane; (**b**) activation of p53 protein pathway; (**c**) activation of apoptotic proteins; (**d**) mitochondrial membrane leakage; (**e**) release of Cyt-C proteins; (**f**) activation of CaSpase3; (**g**) cleavage of nucleus membrane; (**h**) fragmentation of DNA strands; (**i**) upregulation of apoptosis inducer genes; (**j**) downregulation of anti-apoptotic gene; and (**k**) cell zeiosis (blebbing).

Based on the above facts, attachment of nanoformulation loaded with a microalgal-based molecule to the cell membrane can result in the damage of membrane integrity, thus triggering the activation of p53 protein. As a result, p53, a known activator of pro-apoptotic genes, activates Bax, Bad and Bak proteins, which are known to cause mitochondrial membrane leakage and release Cyt-C, thereby activating caspase-3 during the cascade reaction. Eventually, caspase-3 cleaves the nuclear membrane to induce DNA fragmentation. At the same time, up-regulation of C-myc, a known inducer of apoptosis, contributes further amplification of the apoptotic signals and down-regulation of anti-apoptotic genes, Bcl-2 and Bcl-XL corroborating manifestation of apoptosis and consequent cell blebbing (Figure 4).

Recently, Ahmed et al. [59] isolated the cyanobacterial species *Fischerella* sp., from the Nile river in Egypt, and its bioactive compounds were found to be active against liver cancer (HepG-2), lung cancer (A549), colon cancer (HCT116), and breast cancer (MCF-7) cells. *Fischerella* BS1-EG crude extract exhibited various effects on all tested cell lines. GC-MS analysis detected 29 different compounds, including fatty acids, alkaloids, phenols, and amino acids, which were found effective as anti-cancer agents.

## 3. Current Trends in Toxicity and Safety Concerns of Microalgae/Cyanobacteria

A main topic of interest in the field of natural biomedicines and their nanoformulations is toxicants or toxicity level. Due to the characteristic physicochemical properties of various microalgal systems and targeted nanoformulations, unpredictable health outcomes of microalgal nanoformulations have been important to scientists, suggesting they exclusively tackle the toxicity issues related to microalgal-based nanoformulations for their safe used in medicine development [60]. 

Several mineral elements are essential for living organisms at very low concentrations, but at high concentrations, most of them are toxic and have a direct and adverse influence on various physiological and biochemical processes [61,62]. Taneva et al. [63] reported that freshwater *Phormidium* species (microalgal species) could be considered as an environmental risk as well as a source of therapeutic agents. Over recent years, global incidences and frequency of algal toxicity have increased with occurrence of human intoxication by a variety of algal sources, suggesting that species associated with the cyanoprokaryota group could be the sources of toxin production [34]. 

Blue-green algae products are free of contaminants, however, liver-damaging substances such as microcystins, toxic metals, and harmful bacteria, are unsafe for human consumption [64]. As demand for marine algal products has increased, annual safety assessment of blue-green algae products has found them to be contaminated and unsafe for children [65]. Contaminated blue-green algae can cause liver damage, stomach pain, nausea, vomiting, weakness, thirst, rapid heartbeat, shock, and death. Research on microcystins reports high toxicity [65].

Β-methilamino-L-alanine (BMAA), a non-proteinogenic amino acid, is found in types of microalgae, including cyanobacteria, which are usually found in waterways, damp soil, and on the roots of cycad plants [65]. Normally, blue-green algae occasionally cause algal blooms, when there is a rapid growth of organisms due to high levels of nutrients in the water. The resulting bloom can sometimes become so large that it can be toxic to wildlife. Currently, several marine researchers reported that when high concentrations of BMAA were present in neuron-like cells, it transformed into amino acid L-serine during protein formation, creating a faulty protein within cells [65]. The faulty proteins were found to change shape so that they could no longer perform their role within cells, causing them to clump together. The researchers also observed that cells died once BMAA was substituted into the protein [65].

A study reported by the *Los Angeles Times* showed that toxins from algal blooms may cause Alzheimer’s-like brain changes [65]. Chronic exposure to an environmental toxin may increase the risk of neurodegenerative illness. After an unusual illness suffered by Chamorro villagers on the Pacific island of Guam, researchers have been investigating BMAA for more than 40 years. However, this was considered as controversial since earlier research showed that microalgae were promising for treatment of Alzheimer’s disease (AD) [65]. In a recent study, El-Baz et al. [66] reported that *Dunaliella salina* extract protects against AD and possesses a variety of activities, including antioxidant effects due to its ability to create large amounts of carotenoids. Considering these facts the matter is still a controversial platform or anticipated only for a few toxin cases which might be from the effect of over production of algal blooms and BMAA.

In addition, previous studies have shown severe concerns on five groups of photosynthetic cyanobacteria and algae-based toxins due to their serious impact on human health that include neurotoxins (anatoxins and saxitoxins), cytotoxins (cylindrospermopsin), hepatotoxins (nodularin and microcystins) dermatotoxins and irritant toxins or endotoxins (lypopolysaccharides and lipopolysaccharides) [67]. A plethora of reports have been published on toxin producing ability of microalgae [68]. Hinder et al. [69] described toxic syndromes of microalgae based on their toxicity profile, ability to produce toxins, and epidemiological aspects. A few important microalgal toxins such as saxitoxin and gonyautoxin from *Alexandrium* and *Gymnodinium* spp., respectively; domonic acid from *Pseudo nitzschia*, and yessotoxin from *Gonyaulax spinifera*, *Lingulodinium polyedrum*, and *Protoceratium reticulatum* were reported for different toxin syndromes and symptoms within UK water, including tingling and numbness, drowsiness, nausea, diarrhoea, abdominal cramps, and dehydration [69]. 

## 4. Status of Microalgal Anti-Cancer Drugs in Commercialized Platforms

Natural therapeutic agents derived from marine resources have generated significant contribution in the development of anti-cancer drugs with a public approval of clinical trials especially in the USA and Europe. The last decade of marine drug discovery has reviewed the importance of marine-based compounds for their successful clinical trials [70]. In spite of failures on the approval of drugs for Phase I and Phase II clinical trials due to their severe anaphylaxis and neuromuscular toxicity, a paradigm has been set on isolating anti-tumor/anti-cancer compounds from marine microalgae based on their significantly greater potential than conventional anti-tumor agents. With a view on the nutritional point to human health, cyanobacterial species of *Spirulina* could be consumed directly without any pre-processing step. They have been shown to augment the immune system and possess anti-cancer effects, thereby providing defense to cells against a variety of disorders such as inflammation and cancer [71,72].

Efficacy and safety of a series of anti-tumor compounds must be determined at the biomedical platform followed by assessment of their adversary side effects using clinical trials. Also, their therapeutic index should be measured so as to determine their efficacy in cancer therapy. Contrary to these, a number of clinical trials are terminated, if the target compound is non-specific, and has the ability to destroy significant amounts of normal healthy cells than cancer cells. Different types of cancer cells exhibit a wide range of properties, thus determining their tolerance ability to the drugs. Individuals may not be sufficiently tolerant to an increased dose of the drugs, and this may lead to terminate the drug in going for clinical trial approval [73].

Four principal components can explain 87% of the variation found among 343 bioactive natural products and 96 FDA-approved anti-cancer drugs [74]. Across the four dimensions, cyanobacterial isolates occupy similar and distinct areas of chemical space that collectively align well with FDA-approved anti-cancer agents, thus, examining this separate resource for anticancer drug development.

A variety of bioactive natural products are being produced from microalgae with a significant commercial and biomedical potential. Nonetheless, only a few of them are produced at industrial level, due to their low production and difficulty in isolating them economically [75,76]. Scientific efforts in this area have been undertaken to select high-yield strains, to optimize cultivation, and to use a genetic engineering approach to modify the strains to obtain high value-added products [77]. 

Pertaining to these, chemicals which have direct ability to modulate or trigger cell metabolism have been proposed and applied in a number of microalgae with significant commercial value [78]. However, further efforts with significant strategies are still needed to establish more ideal and optimal conditions in order to obtain higher amounts of bioproducts. These products derived from microalgae such as fatty acids, carotenoids, proteins, polysaccharides, and phenolics have shown enormous biomedical and pharmaceutical potential based on their sustainable anti-inflammatory and anticancer properties [8].

Two new 2-alkypyridine alkaloids, such as phormidinine A and phormidinine and B were isolated from a marine cyanobacterial species, *Phormidium* sp. [79]. A series of polychlorinated acetamides and their dechlorinated derivatives having terminal mono-, di-, or trichlorinated functional groups were reported from *Microcoleus lyngbyaceus* and *Lyngbya majuscula*/*Schizothrix assemblage*, respectively, including some other marine cyanobacterial metabolites, such as, dysidenin-type compounds and barbamide [80]. Additionally, taveuniamides have been reported from cyanobacterial species *L. majuscula*/*S. assemblage* [80]. Furthermore, two antibody drug conjugates pinatuzumab vedotin and tisotumab vedotin were reported from mollusks and cyanobacteria, which based on their efficacy against non-Hodgkin lymphoma and chronic lymphocytic leukemia [81] and ability to inhibit tumor growth [82], respectively, have been approved for Phase I clinical trials.

There are not many commercialized marine-based drugs available on the market. However, research has been published to prove their anti-cancer effects. The pipeline of marine-based anti-cancer drug development is quite dynamic with a significant and growing number of marine-derived compounds entering into clinical trials, although some others have been discontinued for several reasons. The prime reasons for withdrawal of approval from clinical Phase I to Phase III are lack of efficacy (43%) and drug toxicity (33%). Table 1 represents a list of a few well-commercialized marine-based anti-cancer drugs. There are only one or two cyanobacteria-based drugs available on the commercial market, despite numerous published compounds from cyanobacteria for their anti-cancer effects. 

Biodiversity focuses on challenges associated with safe access to marine resources along with proper identification of the biological material and the efficient screening of samples and compounds. Supply and technical category deals with the challenges associated with the actual process of isolation and sustainable production of the pure bioactive compounds, as well as understanding of their mechanism of action towards the desired target. Finally, market category considers the process and costs of developing natural bioactive products [83]. These topics must be considered when developing strategies for microalgae-based efficient anti-cancer drug discovery and their commercialization in the medicine sector.

## 5. Strategies to Address Difficulties: Development of High Potency Drug Delivery Formula

### 5.1. Possibility of Peptide-Based Microalgal Drug Development

Before establishment of a commercialized platform to overcome the difficulties associated with natural compound-based drug development such as stability and solubility, a peptide conjugation strategy was adopted in the pharmaceutical sector [84]. Natural peptides from different sources and their synthetic analogs have been the basis for a number of studies performed to discover new therapies for treating malignant cells [85]. Various cationic anti-tumor peptides have been suggested as promising agents for anti-tumor therapies due to their numerous advantages over other chemical agents such as low molecular mass, relatively simple structure, greater specific cytotoxicity to tumor cells over healthy cells, fewer adverse reactions, ease of absorption, various routes of administration, and low risk to induce multi-drug resistance [86,87,88,89,90]. While using them in combination with other conventional therapeutic agents, this may result in the enhanced drug efficacy of the treatment agent [84]. Still, several cancer drugs are in research progress with peptide-based conjugational anti-cancer drug development. 

Currently, approximately 50–60 approved peptide drugs are available in commercialized platforms, and this number is expected to increase in future. A report detailed that four peptide drugs on the market reached global sales of over $1 billion USD, and three of the peptides are currently used in treating cancer: leuprolide acetate (Lupron), goserelin acetate (Zoladex), and octreotide acetate (Sandostatin) [85,91]. Approximately 30 peptides were discovered/developed as peptide-based anti-cancer drugs, in order to provide a basic platform in cancer therapy [92]. On the whole as a better alternative of a single moiety of the microalgal isolated compound, the combinational effect with peptide formulation could overcome the barriers with the advantages of low cost scenario of marine-based drug developments in pharmaceutical commercialization. 

There are a few limitations of these peptide-based developments, including poor bioavailability, instability, and insolubility with natural compounds, and they show toxicity to host cells [91]. Cost effective production processes with marine compounds may cause major limitations for commercialized platforms. Therefore, researchers are moving to a diversified field of nanoformulations for cost effective and rapid developments.

### 5.2. Possibilities and Facts Related to Nano-Formulated Microalgal Drug Development

Nanoparticles are classified into organic (carbon-based nanoparticles) and inorganic nanoparticles. Magnetic nanoparticles (iron), noble metal nanoparticles (silver, gold, and platinum), and semiconductor nanoparticles (cadmium, zinc oxide, zinc sulphite, silica, and titanium oxide,) are grouped as inorganic nanoparticles. Indeed, the special properties of nanoparticles, such as biocompatibility, large-scale production, simple usage, and functional efficiency, make them effective in drug delivery [93]. Gold (Au) and silver (Ag) nanoparticles have multiple applications in diverse fields [94]. Nanoparticles have a wide range of applications in the field of cancer drug delivery and diagnostics [94,95]. Of various metal nanoparticles (Ag, Au, Pt, Pd, and Cu), Ag nanoparticles have attracted much attention in the field of nanotechnology due to their distinct physicochemical and biological properties [95]. 

To overcome the limitations to the use of peptide-based drugs, nanonization of natural product-based medicines has attracted huge attention due to their poor solubility and stability. It was reported that nanotechnology is one of the fastest platform for developing nanoformulations [96]. Researchers are synthesizing safe nanomaterials including metals, lipids, polysaccharides, and protein- or peptide-based nano-formulated drug targets. Nanoformulations have increased particle size and surface area due to increases in bioavailability, stability, and solubility, and reduce the side effects of natural compound-based drugs. They are also useful for the treatment, diagnosis, monitoring, and control of biological systems and have recently been named as a nanomedicine [94].

In recent years, Elghazawy et al. [96] tested the enhanced anti-cancer potential and improved solubility of new quinoline derivatives by using a nanoformulation strategy. As a result, synthesized nanomicelles of new quinoline derivatives showed observable significant increases in cytotoxic efficacy, demonstrating a positive impact of the used nanoformulations on the delivery of active compounds to their cellular biological targets. These facts have impressed researchers working with microalgal compound-based drugs.

Marine bionanotechnology is an innovative area of research with a growing concern. Thus, concerning marine biotechnological prospects, Singh et al. [97] reviewed the information on marine-based synthesis of nanoparticles, their biomedical applications, and mechanisms associated with them. Recently, a range of marine resources have been extensively utilized in the fields of nanoscience and nanotechnology [97]. In various marine bioresources, nanostructures of 1–100 nm size are detected such as seashells, pearls, and fish bones [97].

Recently, Ibrahim et al. [98] developed marine krill lipid-based liposome nanoparticles referred to as “marinosomes”, which encapsulate curcumin into krill lipid-based liposome nanoparticles, and developed a stable anti-cancer nanoformulation from low-cost and readily available nutraceuticals. These “marinosomes” are marine krill lipid-based liposomes containing a high ratio of marine polyunsaturated fatty acids (PUFAs) [99]. Marine PUFAs are divided into two subgroups: omega-6 (n-6) and omega-3 (n-3), which are metabolized and stored in cell membrane phospholipids [100]. They affect membrane fluidity, regulate a wide range of functions in the body, and correct development and functioning of the brain and nervous system [101]. In marine lipids, which are extracted from marine organisms such as krill, a large portion of n-3 PUFAs is bound to phospholipids and contains a higher content of n-3 long chain PUFAs such as eicosapentaenoic acid (EPA; 20:5 n-3) and docosahexaenoic acid (DHA; 22:6 n-3) [102,103]. A likely mechanism of cancer prevention based on the anti-inflammatory properties of n-3 PUFAs has been reported [104]. Other studies have reported different mechanisms of cancer prevention by n-3 PUFAs, including apoptosis, inhibition of angiogenesis, inhibition of metastasis, and others [105]. Previously, Anand et al. [106] synthesized monodispersed and spherical silver nanoparticles of 20–60 nm from marine fungal species (*Aspergillus flavus* SP-3, *Trichoderma gamsii*, *Talaromyces flavus*, and *Aspergillus oryzae*) and reported their anti-cancer effects.

Ramkumar et al. [94] reviewed the literature on marine algae and reported marine algae as a rich source of marine biomolecules, such as carotenoids, proteins, polysaccharides (alginate, laminaran, and fucoidan), amino acids, vitamins, polyphenols, and minerals. Marine algae can be used in food, medicine, and manufacturing. However, there is not sufficient published data on nano-formulated medicine development from microalgal resources (Table 2). Considering the lack of information on microalgal nano-formulated drugs for cancer therapies, there is a need for the pharmaceutical sector to develop a variety of effective nanoformulations.

### 5.3. Interest in Albumin-Based Nanoparticles: Useful Tool for Nanoformulation

Biosafety is the major concern in the clinical translation of nanomedicines. In recent years, efficacy of albumin has been confirmed as a valuable material for the production of nanoparticles, especially in the fields of bioimaging and drug delivery [112]. A considerable interest has been shown by biomedical practitioners on precise diagnosis and effective drug delivery in cancer therapy [113]. Additionally, nano-systems with effective drug delivery have shown significant potential in cancer treatment by targeting prolonged blood circulation time [114]. Moreover, nanocarrier materials posing adversary side effects have encouraged researchers to investigate relatively more biocompatible materials for the construction of nanoparticles [115]. Successful application of albumin for using abraxane in clinical nanomedicine has proved its significant potential to be used as the most effective nanocarrier for the development of novel nanomedicines [116]. Mostly two types of albumins are used for the construction of nanoparticles: human serum albumin (HSA) and bovine serum albumin (BSA). Both types of albumins are serum albumin proteins (human and cow) and share many of the same properties, including high water solubility, long half-life in blood, similar molecular weight (65–70 kDa), and similar numbers of amino acid residues (585-HSA and 583-BSA) [117,118]. No observances have been noticed regarding the properties of HSA and BSA while constructing a nanomedicine. Hence, both types of albumins have shown significant applications in designing multifunctional nanoparticles. The developed strategies with alternative albumin could easily be replaced, therefore, here we have discussed both types of albumins and given a comprehensive summary on the developing technologies of albumin-based nanomedicine. A number of reports have been published in literature on albumin-based nanomedicine from different aspects, including preparation technologies, hybrid albumin nanomedicine, half-life extension, bioconjugate chemistry, and theranostic applications [119,120]. Although these reports have well summarized the efficacy of albumin-based nanomedicine and prompted the quick development of the technologies in this field, none of them focused on the roles of albumin in formulating multifunctional nanoparticles. The unique multi-properties of albumin can give significant opportunities to construct multifunctional nanoparticles with the expected properties.

Albumin hydrophobic pockets have a significant role in effective drug delivery by facilitating albumin binding to hydrophobic or amphiphilic small molecules [119]. The binding between albumin and drugs/peptides could be achieved by direct interactions between albumin and drugs/peptide or interactions between albumin and prodrugs/peptide-conjugate. The albumin and drugs/peptide bind together when there are strong interactions between them [121]. The resulting prodrug/peptide-conjugate could form a stable nanocomplex with albumin. This nanocomplex while in a body-fluid environment can obtain excellent stability, thus confirming its practical application in a difficult in vivo system [122]. Even though the commercial value for albumin is very high, the study of site-selective modification of albumin is still rare. We believe that the accumulated knowledge in the site-selective antibody modification will prompt the development of site-selective albumin modification and give functionalized albumin nanomedicine with higher reliable quality. This interest could also be applicable in the case of marine microalgal nanoformulations for improved commercialization.

### 5.4. Microalgal Polysaccharide-Based Nanoformulation

Marine algae are excellent resources for the production of sulfated polysaccharides, which have received increased interest in the nutraceutical, cosmeceutical, and pharmaceutical industries [123]. Moreover, a huge amount of attention is being paid by nano-biotechnologists on polysaccharide-based nanomaterials owing to their excellent biocompatibility and stability, high biodegradability, low cost efficacy, non-toxic character, and unique physicochemical properties as novel nanocarriers for bioimaging and therapeutic applications [124]. These properties are of special interest in the field of nanotechnology and make marine polysaccharides promising as biomaterials. In addition, several researches have investigated polysaccharide-based nanomaterials for biomedical applications such as anti-microbial activity, drug delivery, gene delivery, tissue engineering, cancer therapy, and wound healing [125,126].

Researches have highlighted that polysaccharides released into culture medium by cyanobacteria present several biological activities and anti-viral effects in the human body [127]. Cyanobacterial polysaccharides released into medium by *Arthrospira platensis* exhibited anti-viral activity in vitro and in vivo against two strains of vaccinia virus and *Ectromelia* virus. Moreover, a novel acidic polysaccharide, nostoflan, derived from *Nostoc flagelliforme* exhibited remarkable anti-viral potential against a variety of enveloped viruses such as influenza virus [127]. Free radical scavenging compounds such as sulfated polysaccharide isolated from cyanobacteria can also be used to reduce cancer formation in the human body. Further, polysaccharides extracted from *Spirulina* for medical application have shown various biological activities [128]. Sulfated polysaccharides from *Spirulina* inhibit the proliferation of tumor cells in vitro [129] and in vivo [130]. In addition, some of the species of *Chaetoceros, Isochrysis, Nannochlropsis, Pavlova, Phaeodactylum, Skeletonema, Thalassiosira* and *Tetraselmins* have shown potential application in animal nutrition [131] due to the abundance of biologically active contents of essential lipids, such as omega-3 and omega-6 fatty acids [132]. Although microalgae differ in composition, components derived from them may have a better application when formulated with other nanostructures. 

**Figure 5:**
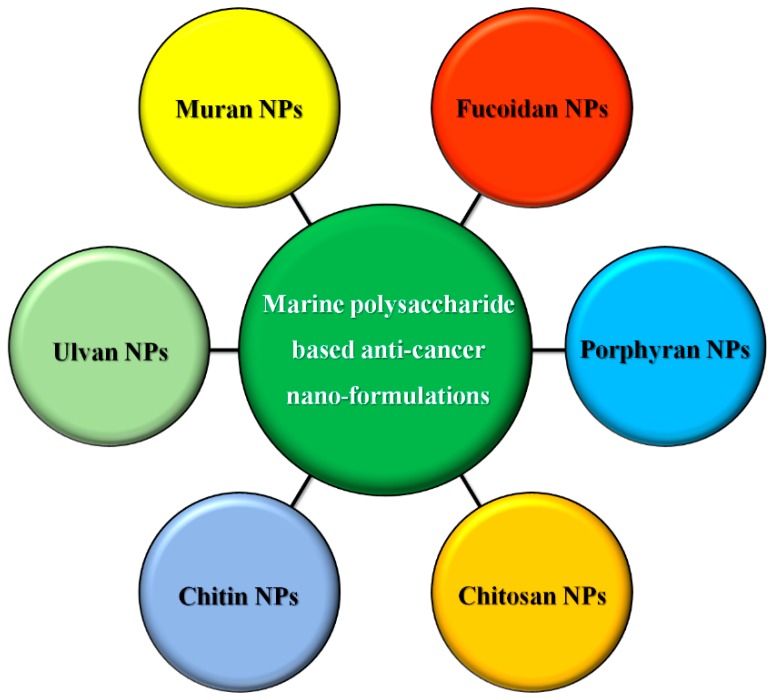
Marine originated polysaccharides and their applications in cancer therapies in combination with nanobiotechnology. NP: Nanoparticle.

Based on a literature survey, several reports have advocated that polysaccharides originating from microalgae especially sulphated polysaccharides have the potential to inhibit tumor growth by preventing tumorigenesis through oral consumption [133]. Based on the above-mentioned facts, anticancer strategies can be designed for a few microalgal sulfated polysaccharide-based nanomaterials against cancer and other synergistic microalgal nanoformulation developments and their nano-form commercialization (Figure 5).

## 6. Facts of the Commercial Market

Although the current review focuses on the developing facts and benefits of microalgal nanoformulations and their awareness in medicine sectors, there is still a lack of microalgal related nano-drug formulations especially for cancer targets. However, some kinds of microalgal or cyanobacterial secondary metabolites, and photoprotective compounds have been applied in the form of nanoformulated anti-aging creams, antioxidants, and anti-inflammatory creams/drugs isolated from these photosynthetic microorganisms [134,135,136,137]. The effective compounds, against human diseases, including cancer isolated from microalgal species need attention for their use in nanomedicines for effective commercialization.

Some aquatic organisms such as *Dunaliella salina* (green algae), *Ascophyllum nodosum* (brown algae), *Chlorella vulgaris* (green algae), *Chondrus crispus* (red algae), *Spirulina platensis* (blue-green algae), *Mastocarpus stellatus* (red algae), *Alaria esculenta* (brown algae), and *Nannochloropsis oculata* (algae), have obtained significant positions in the skin treatment field, including skin related cancer disorders [138], but only few kinds of algal nanoformulations are on the commercial market.

In the past few years, cases of non-melanoma skin cancer (NMSC) have increased [139], and use of nanoformulated sunscreen with effective non-toxic nanocarriers is considered beneficial in these cases by health care professionals [139,140]. For example, effective nanoformulation of micosporin amino acids (MAAs) and scytonemin from cyanobacterial sources has been tried and used as an efficient and natural UV blocker and protective medicine against skin cancer disorders in the form of nano-medicines. Efficacy of these MAAS has been confirmed in preventing both UV radiation damage and other skin cancer associated disorders [141]. 

Recently, the National Cancer Institute (NCR) declared anti-carcinogenic effects of a fat-soluble and cyanobacterial-based photosynthetic pigment, β-carotene with an excellent efficacy in reducing risk of heart disease by controlling the cholesterol level, thus confirming its superiority as an anti-carcinogenic agent with an ability to control heart-associated diseases [134]. Due to these desirable medicinal properties, there is an increasing demand for natural β-carotene for its biomedical applications. *Arthrospira* sp. as a rich source of γ-linolenic acid (GLA), is a very important cyanobacterial species, which has shown enormous potential in regulating lipid metabolism, thereby it has the ability to reduce high blood pressure [134]. A variety of biologically active molecules (enzymes and antibiotics) with significant anti-inflammatory and anti-cancer properties have been reported from various cyanobacteria and algae [142,143], which suggests that the compounds derived from these photosynthetic microorganisms may be important for their biotechnological applications.

In a recent survey, Jasparas et al. [144] focused on marine biodiscovery and research pipeline for future ocean medicines, and discussed a case study of a European Union-funded project, PharmaSea, which aims to discover novel products for the treatment of infections, inflammation, and neurodegenerative diseases. Currently, there are about 26 natural products in Phase I to Phase III clinical trials, twenty-three as anticancer cancer agents, two for schizophrenia and Alzheimer’s, and one for chronic pain [144]. Thus, the pipeline of promising marine derived compounds is very strong, and several of these agents are likely to reach the commercial market in the coming years [70].

A supporting news also proved that in recent years scientists transformed algae-based compounds into cancer killing nano-drug delivery systems; they developed a carbon based nano-delivery system with microalgal combination, *Thalassiosira pseudonana*, a common type of photosynthesizing algae, effectively killing targeted cancer cells without any adverse effect on normal and healthy cells [145]. 

Based on the above literature, it could be anticipated that microalgae-based nanoformulated delivery systems are in urgent need to be commercialized in the current scenario of various cancer related diseases. However, until now in the literature, the aforementioned aspects remain unexplored for commercial nanoformulations or stable, effective formulations of microalgae drugs and their proper usages in nanomedicine-based therapies. It could be interesting to try the use of diatoms and other microalgal species for commercialized nanoformulations in cancer therapies without risk of having any side effects. Research in this sector will open new doors for harnessing the potential of marine microalgal natural products with nanoformulated pharmaceutical properties.

## 7. Concluding Remarks and Future Perspectives

Since the beginning of civilization, biologically active compounds, which are obtained from a diverse range of algal and cyanobacterial community, have been widely used in a variety of industrial and biomedical aspects. Cyanobacteria and algae are rich bioresources of multitudes of biomolecules, including polysaccharides, pigments, essential lipids, polyketides, vitamins, lectins, steroids, antioxidants, fibers, MAAs, proteins, and halogenated compounds. These compounds are widely used in the fields of nutraceuticals, pharmaceuticals, and biomedical due to their multifunctional applications. Undoubtedly, our understanding in the field of algal metabolites has significantly improved in the past decade, but there are still many challenges to their application in the medicine sector and for their nanoformulation and commercialization. Uncovering novel enhanced anti-cancerous mechanisms of nanoformulations consisting of algal secondary metabolites will add to the development of nanomedicine and marine drugs.

**Figure 6:**
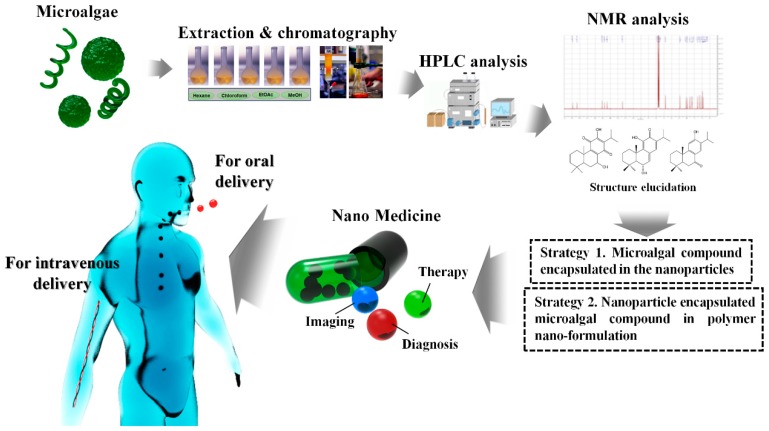
Overall concept for scale-up microalgal nanoformulations; the way to a commercialized platform for oral and intravenous drug delivery in anticancer drug developments.

In the current review, we emphasized the lack of commercialized drug development using microalgae and their structurally diverse biologically active compounds such as terpenes, alkaloids, steroids, polysaccharides, lipids, and polyphenolics. Additionally, further technologies are needed to explore the effectiveness of these anti-cancer secondary metabolites as nanoformulations with nanoparticles. Nano-fusion technology and green technology should be fused to exploit these cyanobacterial and microalgae anti-cancer drug nanoformulations. A concept from beginning to end has been drawn to catch the reader’s interest for further future researches to achieve high innovation in commercialized platforms (Figure 6). Scale-up commercialization with technology development should also be a major task for cost effective marine microalgae nanoformulations. Future works should explore the novel functions of microalgae secondary metabolites.

## Figures and Tables

**Table 1:** Major commercialized and development phase of marine-based anti-cancer compounds/drugs.

Natural Compounds/Drugs	Source	Company Launched	Status after 2015 Food and Drug Administration (FDA)/European Medicines Evaluation Agency (EMEA)
Brentuximab vedotin 63 (Adcetris™)	Cyanobacteria: *Symploca hydnoides* and *Lyngbya majuscula*	Seattle Genetics (Bothell, WA, USA)	In market with antibody-drug conjugates
Glembatumumab vedotin	Cyanobacterium: *Lyngbya* sp.	Celldex Therapeutics	Phase II
DMMC (Cyclic depsipeptide)	Cyanobacterium: *Lyngbya majuscula*	-	Preclinical
Largazole	Cyanobacterium: *Symploca* sp.	-	Preclinical
Apratoxin A	Cyanobacterium: *Lyngbya boulloni*	-	Preclinical
Cryptophycin 1	Cyanobacterium: *Nostoc* sp. GSV 224	Merck Pvt.	In market
Tasipeptins A–B	Cyanobacterium: *Symploca* sp.	-	Preclinical
Coibamide A	Cyanobacterium: *Leptolyngbya* sp.	-	Preclinical

**Table 2:** Nanoformulations from marine resources for anti-cancer therapies.

Marine Resources	Name of the Species	Nanoparticles/Size (nm)	Activity	References
Seagrass	*Cymodocea serrulata* (Aqueous extract)	Ag/5–25	Anticancer	[107]
	*Cymodocea serrulata* (Aqueous extract)	Ag/17–29	Anticancer and cytotoxicity	[108]
Salt marshes	*Suaeda monoica* (Leaf extract)	Ag/30–31	Anticancer	[109]
Sand dune	*Citrullus colosynthis* (Callus extract)	Ag/85–100	Anticancer	[110]
Marine fungi	*Aspergillus flavus*, *Trichoderma gamsii*, *Talaromyces flavus*, and *Aspergillus oryzae* (Cell-free filtrate)	Ag/20–60	Anticancer	[106]
Marine mussel	*Mytilus galloprovincialis* (Muscle tissue)	Cadmium-based quantum dots/6–10	Immunocytotoxicity, cytogenotoxicity and genotoxicity	[111]
Marine cocktail	Marine poly unsaturated fatty acids (PUFAs) (Curcumin)	Lipid nanoparticles/100–200	Anticancer	[98]
